# Mastocytosis: from a Molecular Point of View

**DOI:** 10.1007/s12016-017-8619-2

**Published:** 2017-07-19

**Authors:** Daniel Elieh Ali Komi, Todd Rambasek, Stefan Wöhrl

**Affiliations:** 1 Immunology Research Center, Tabriz University of Medical Sciences, Tabriz, Iran; 2 Department of Immunology, Tabriz University of Medical Sciences, Tabriz, Iran; 3 Ohio University Heritage College of Osteopathic Medicine, Athens, OH USA; 4 Floridsdorf Allergy Center (FAZ), Vienna, Austria; 5 Department of Dermatology, Division of Immunology, Allergy and Infectious Diseases (DIAID), Medical University of Vienna, Vienna, Austria

**Keywords:** Cutaneous mastocytosis, D816V mutation, KIT, Mast cell, SCF, Systemic mastocytosis

## Abstract

Mast cells (MCs) are physiologically activated by binding of stem cell factor (SCF) to the extracellular domains of the Kit receptor. This binding increases the proliferation and prolongs the survival of normal mature MCs, as well as intensifies the release of mediators. In mastocytosis, somatic mutations of the coding Kit gene cause autocrine dysregulation and lead to constitutive KIT activation even in the absence of its ligand SCF. Clinical symptoms are caused by MC-mediator release and/or infiltration of MCs into tissues. Aberrant KIT activation may result in increased production of MCs in the skin and extracutaneous organs. Depending on the affected organ(s), the disease can be divided into cutaneous mastocytosis (CM), systemic mastocytosis (SM), and localized MC tumors. The updated classification of WHO discriminates between several distinct subvariants of CM and SM. While the prognosis in CM and indolent SM (ISM) is excellent with (almost) normal life expectancy, the prognosis in aggressive SM (ASM) and MC leukemia (MCL) is dismal. The symptoms may comprise urticaria, angioedema, flush, pruritus, abdominal pain, diarrhea, hypotension, syncope, and musculoskeletal pain and are the results of MC infiltration and mediator release into target organs, i.e., the skin, gastrointestinal tract, liver, spleen, lymph nodes, and bone marrow. Mastocytosis differs from a lot of other hematological disorders because its pathology is not only based on the lack of normal function of a specific pathway or of a specific cell type but additionally is a proliferative disease. Currently available treatments of mastocytosis include symptomatic, antimediator and cytoreductive targeted therapies.

## Introduction

Mast cells (MCs) are normal residents of mucosal tissues, but their numbers and anatomical location change markedly during immune responses, infections, and other disorders [1]. In most settings, MCs have become infamous for their detrimental actions, i.e., anaphylaxis, allergy, arthritis, atherosclerosis, and cancer while in some settings, notably host defense against bacteria, parasites, and envenomation, their biologic function is in favor of maintaining health [2]. MC progenitor cells express the tyrosine kinase receptor KIT (CD117). Normally, the interaction between this oncogenic receptor and its ligand, stem cell factor (SCF), induces MC development in uncommitted and MC-committed hematopoietic precursor cells [3]. Mastocytosis is a heterogeneous group of disorders involving MCs and their CD34+/CD117+ progenitors. It is a group of rare clonal disorders of bone marrow origin characterized by abnormal growth and/or accumulation of clonal MCs primarily in the skin and bone marrow. Neoplastic MCs expressing CD25 and/or CD2 were described in systemic mastocytosis especially in aggressive systemic mastocytosis (ASM) and mast cell leukemia (MCL) [4, 5]. The symptoms of mast cell activation include sudden onset of flush, urticaria, angioedema, pruritus, abdominal pain, headache, diarrhea, hypotension, syncope, and musculoskeletal pain which are the results of MC mediator release and infiltration into target organs [4]. The heterogeneity of clinical presentation in mastocytosis is a result of MC burden and MC activity [6], the type of skin lesions, the patient’s age at the onset, and the associated hematological disorders. The typical mastocytosis of childhood is usually cutaneous and transient whereas in adulthood the systemic form is more common [7]. MCs respond to surrounding stimuli through the expression of a variety of receptors including FcεRI and KIT (CD117). MCs require stem cell factor (SCF) binding to their surface receptor KIT for homeostasis [8]. In mastocytosis, the presence of mutations within different regions of KIT in the extracellular, transmembrane, juxtamembrane domains, or activating loop interrupts the normal signaling cascade characterized by constitutive receptor activation independent from SCF [9].

## Mast Cell Origin and Development

MCs are innate immune cells known for their role in allergic and anaphylactic reactions. They functionally can be considered a double-edged knife with both good and bad sides. The bad consists in type I allergic immune responses through crosslinking FcεR1 via allergen-bound IgE. On the good side the MC cell plays a protective key role in the battle against some environmental threats such as the venoms of reptiles and insects [10, 11]. In this regard, MC-derived carboxypeptidase plays a key role in degradation of the snake venom toxin safarotoxin [12]. Owing to expression of a wide range of receptors and release of a broad spectrum of mediators, they play a key role in acquired and innate immunity [13]. MCs arise from hematopoietic progenitor cells and mature MCs ordinarily do not circulate in the blood but migrate into peripheral tissues where they acquire their mature phenotype [14]. CD34^+^/CD117^+^ pluripotent progenitor cells from bone marrow origin circulate in the blood as committed precursors and under influence of SCF develop into mature FcεR1^+^ and CD117^+^ MCs in peripheral tissues [15–17]. SCF, which is produced by a variety of cells including fibroblasts and endothelial cells, promotes the recruitment of MC progenitors into tissues, as well as their local maturation and activation. Its receptor, c-Kit (CD117), is a type III tyrosine kinase broadly expressed on mature MCs and eosinophils [18]. In addition to SCF, MC growth and survival modulators include nerve growth factor (NGF) [15], IL-9 [19], CXCL12, IL-3, IL-4, IL-10, IL-33, and TGF-β [1, 14].

## Mast Cell Tissue Homing

**Fig. 1:**
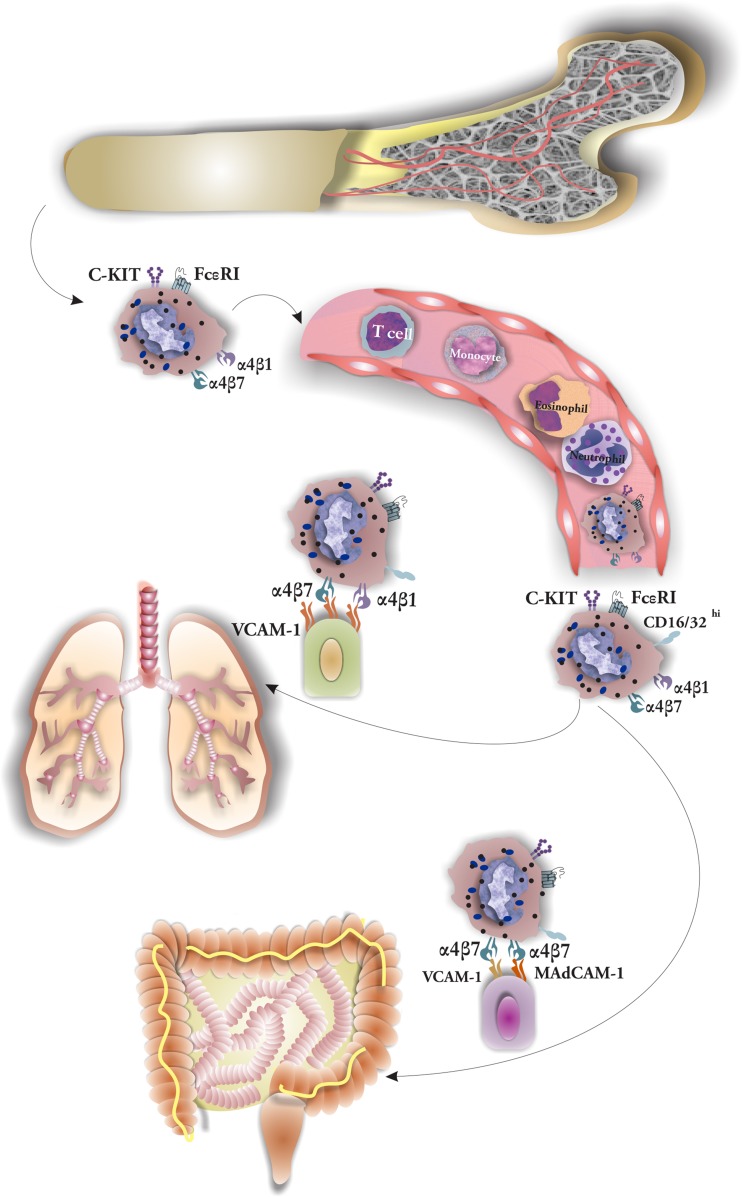
MCs develop from CD34+/CD117+ progenitors of bone marrow origin. Mast cell progenitors (MCP) are released from bone marrow into circulation. There they follow a controlled trafficking pattern with the help of interaction between integrins and their receptors. Finally, they reach the target tissues where under influence of growth factors they mature into mast cells

Tissue homing is a multifaceted process controlled by chemokines and expression of integrin and adhesion molecules both on the surface of progenitors and cells of residing tissues [15, 20]. For instance, trafficking of MC progenitors into the lung tissues requires expression of α4β7 and α4β1 integrins by progenitors and vascular cell adhesion molecule-1 (VCAM-1) by endothelium [8]. α4β7 integrin, expressed on MCs, interacts with (MAdCAM-1) or VCAM-1 on the endothelium and contributes to maintenance of MC number in the small intestine. Moreover, CXCR2, expressed on progenitors, has a role in their directed migration to the small intestine [21] (Fig. 1)

## Mast Cell Mediators and Receptors

**Fig. 2:**
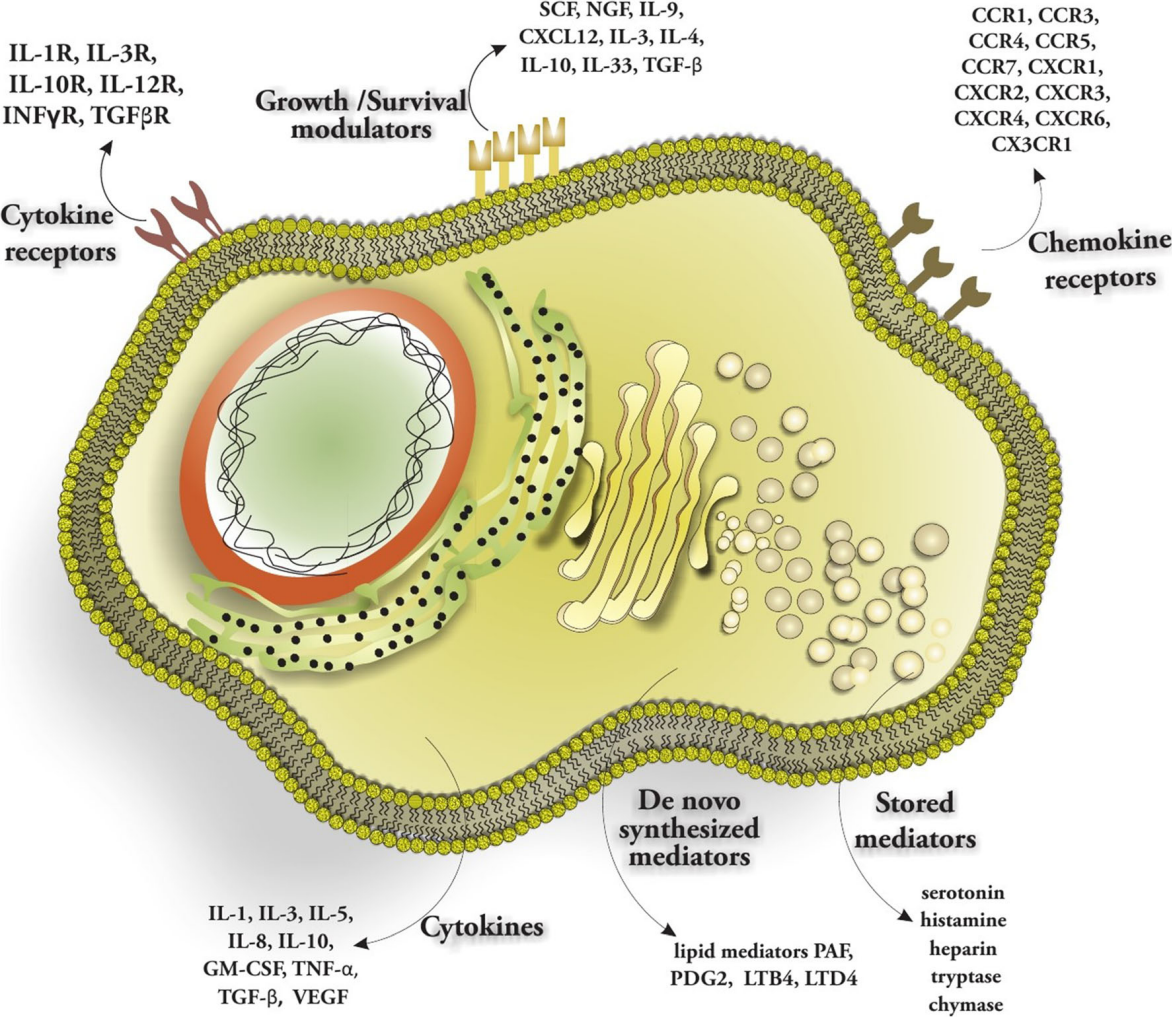
MCs express a variety of chemokine and cytokine receptors on their surface. They release mediators including cytokines, de novo synthesized and stored mediators. MCs rapidly respond to environmental stimuli such as IgE-bound allergen and interact with other immune/non-immune cells

MCs are specialized secretory cells of the innate immune system that play an important role in host defense by producing and releasing proinflammatory mediators, chemotactic factors, and immunoregulatory cytokines. Upon stimulation of their surface Fc receptors with IgE, they immediately release a large number of secretory granules containing histamine, serotonin, and other inflammatory mediators. These mediators play a central role in both the immediate and late-phase inflammatory reaction [22]. MCs produce three categories of effector molecules: (1) pre-formed mediators stored in granules such as serotonin, histamine, heparin, tryptase, and chymase; (2) mediators synthesized de novo upon cell stimulation, among them mainly the lipid mediators PAF, PDG2, and LTB4 and LTD4; and (3) cytokines including IL-1, IL-3, IL-5, IL-8, IL-10, GM-CSF, TNF-α, TGF-β, and VEGF [23]. Human MCs can be categorized into MC_T_ (express high levels of the MC-specific tryptase) and MC_TC_ (express tryptase and chymase) [1, 24]. Morphologically, MCs are characterized by numerous, electron dense cytoplasmic granules containing biogenic amines, enzymes, cytokines, and proteoglycans [25, 26]. They express a wide range of receptors such as FcεRI, Fcγ receptors, complement, cytokine, chemokine, hormone receptors, and toll-like receptors (TLRs). This enables them to secrete a diverse and wide range of biologically active products that enhance as well as suppress immune responses [27] (Fig. 2). Signaling through the high-affinity receptor for IgE immunoglobulins (FcεRI) after binding of type I-allergens is the major pathway for the activation of MCs [28]. However, immunoglobulin free light chains, anaphylatoxins (C3a and C5a) [29], hormones (including corticotropin-releasing hormone (CRH)) [30], and neuropeptides (substance P (SP), hemokinin, neurotensin (NT) and NGF) are alternative ways to activate MCs [13]. After antigen sensitization and specific IgE production by plasma cell, the IgE molecules bind to FcεRI receptors on the surface of tissue MCs and circulating basophils. Re-exposure to the original antigen (or a crossreactive bivalent or multivalent antigen) results in the crosslinking of adjacent FcεRI-bound IgE and the consequent aggregation of surface FcεRI [31]. FcεRI exists in two forms. It can be expressed as a trimeric variant expressed on a variety of immune cells such as monocytes, eosinophils, Langerhans cells, or as a tetrameric variant primarily on MCs and basophils. The tetrameric variant is composed of an IgE-binding α chain, a membrane-tetraspanning β chain that is absent in the trimeric receptor, and a disulfide-linked homodimer of γ chains [32]. The trimeric FcεRI consists of an α-subunit and two γ-subunits. In this form, the α-subunit is a transmembrane protein which binds IgE only insufficiently. The two domains of its extracellular portion adopt the shape of an inverted “v,” the second of which binds one dimeric IgE-Fc molecule asymmetrically through interactions at two sites. IgE binding to FcεRIα results in adopting a unique bent conformation of IgE, and this conformational change contributes to the remarkably slow dissociation rate from FcεRI. The γ-subunit of FcεRI is a transmembrane protein that acts as a common adaptor molecule for various Fc receptors including FcγRI (CD64). The γ-subunit associates as a homodimer formed via a disulfide bond linked between N-terminal cysteine amino acids [33].

## Stem Cell Factor

The knowledge about the molecular structure of the KIT receptor and the further intracellular signaling cascade upon binding to SCF is a prerequisite for a better understanding of the pathogenesis of mastocytosis and is also the basis for treatments targeting this receptor. The discovery of the dimeric molecule SCF (also known as Kit Ligand, Steel Factor, or Mast Cell Growth Factor) and KIT was based on experimental single gene-induced anemias in mice which led to the identification of the W and Steel (Sl) loci. Mutations at either of these loci were shown to cause alterations of fur color, anemia and lack of tissue MCs [34]. Genomic investigations by Geissler et al. revealed that SCF in humans maps on chromosome 12, between 12q14.3 and 12qter [35]. SCF exists both as a membrane-bound and a soluble form expressed by fibroblasts and endothelial cells throughout the whole body [36, 37]. It is synthesized from two alternatively spliced messenger RNAs (mRNAs) as transmembrane proteins which are enzymatically cleaved to produce soluble forms or act as cell associated molecules [38]. Both SCF variants have distinct roles in the survival and proliferation of hematopoietic cells. Using the Sl/Sl^d^ mouse model in which mutants were generated that express only soluble SCF or membrane restricted SCF, it was reported that soluble SCF is responsible for proliferation of myeloid progenitors (in concert with other cytokines), while only the membrane bound form is able partially to correct the runting and the bone marrow hypocellularity that are seen in these mice [39].

## SCF-KIT Interaction and Mediated Signaling

**Fig. 3:**
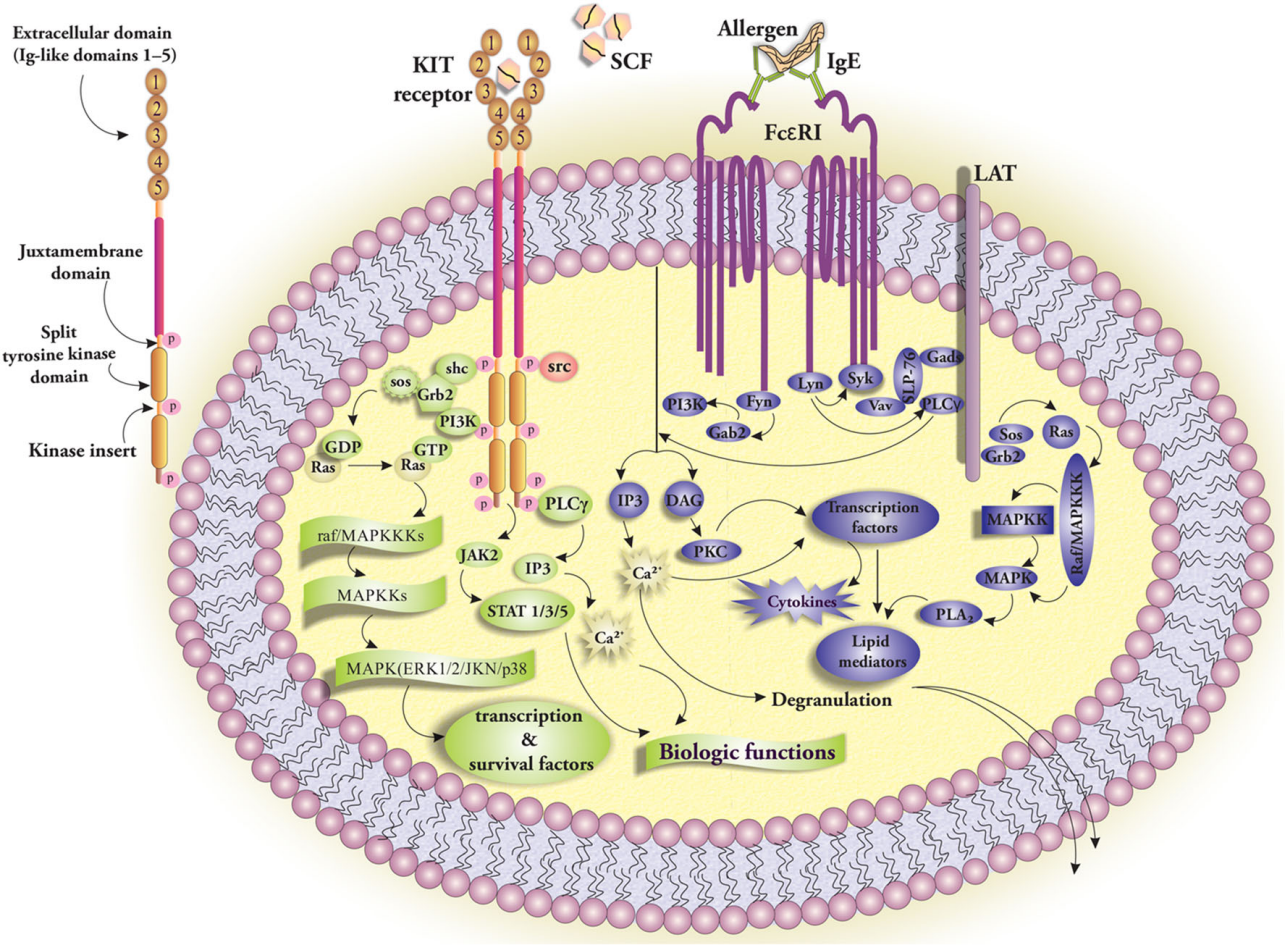
Main signaling pathways in mast cell biology include Kit and FcεRI signaling. While the first pathway (in *green*) is associated with the cell survival and proliferation, the second pathway (in *blue*) has a key role in the production of the delayed de novo mediators and degranulation of the immediately released preformed mediators. SCF binding to the first three Ig-like domains of extracellular region of KIT induces structural changes resulting in dimerization of two KIT receptors following phosphorylation of intracellular regions and activation of signaling pathways. Crosslinking of IgE-bound allergen triggers the FcεRI signaling pathway (*blue*) initiates and results in mast cell degranulation

The c-Kit receptor, encoded by the oncogene c-kit [36], is a type III receptor tyrosine kinase with five extracellular immunoglobulin-like domains followed by a single transmembrane-spanning region. The first three Ig-like domains possess complementary shape and charge and are capable of binding to SCF, while domains 4 and 5 are involved in the KIT receptor dimerization. A juxtamembrane region is located nearly 30 amino acids between the plasma membrane and the kinase domain and forms the first part of the intracellular section contributing to the regulation of c-Kit kinase activity. The kinase domain consists of two subdomains, tyrosine kinase domain 1 and 2, and is interrupted by a kinase insert sequence [40]. SCF-mediated c-Kit signaling plays important roles in mediating angiogenesis, migration, cell survival, and proliferation of MCs [41]. Binding of SCF to KIT leads to homodimerization of c-Kit by interactions between the Ig-like domains 4/5 of two monomeric KIT receptors. Such interactions pave the way for the consecutive transphosphorylation in the regions “juxtamembrane,” “kinase insert,” “kinase domain,” and finally, “COOH-terminal tail” [40]. Phosphorylated residues act as docking sites for signaling molecules such as Src and Shc kinase, phosphoinositide 3-kinase (PI3K), and phospholipase Cγ (PLCγ). GTP exchanger Sos, PI3K, PLCγ, and JAK2 activate the Ras-Raf-Map kinase (MAPK) cascade which results in Ca2^+^-influx and activation of transcription factors required for MC activation [42] (Fig. 3). SCF-induced activation of JAK2 in human MCs results in STAT5 and STAT6 activation. STAT5 activation promotes MC development, survival, and proliferation. Interleukin-3 (IL-3) is crucial for MC expansion and activates JAK2, STAT3, and STAT5. Interestingly IL-3 and SCF share overlapping or synergistic functions in MCs because of their concomitant STAT5 activation [43].

## Molecular Mechanisms

**Fig. 4:**
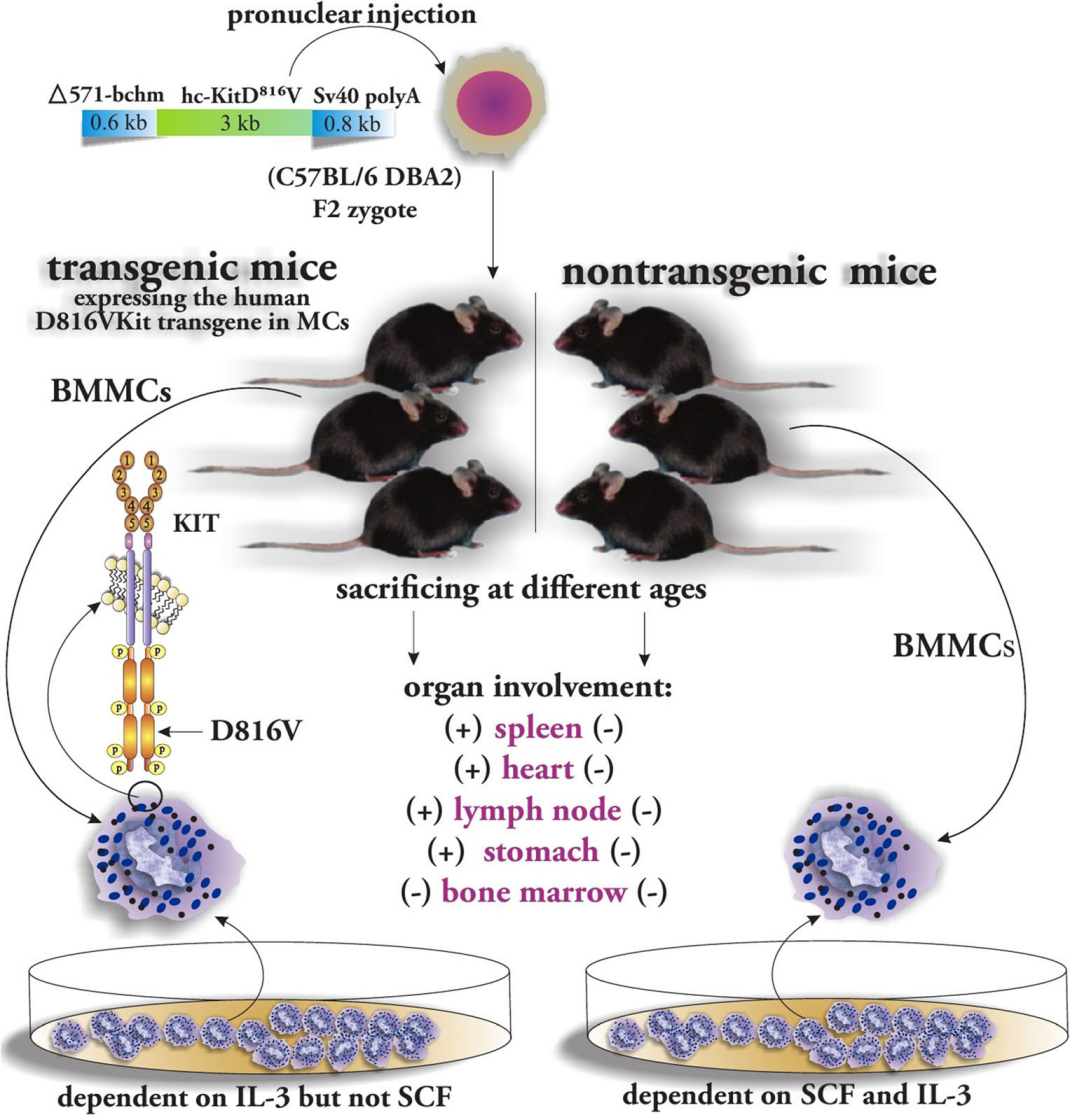
Transgenic mice expressing the human D816VKit transgene in MCs were assessed at different ages for developing SM symptoms and compared with normal non-transgenic mice. They were found to have organ involvement in the spleen, heart, lymph nodes, and stomach but not in the bone marrow. Comparing BMMCs of these two groups of mice revealed that BMMCs from the transgenic ones became independent to SCF for proliferation and activation

Nagata et al. identified a point mutation consisting of a substitution of valine for aspartic acid in the catalytic domain of c-KIT (ASP816VAL or D816V) in the peripheral blood of patients with mastocytosis. A year later, this discovery was followed by identification of the same mutation in CM and aggressive systemic mastocytosis (SM) [42]. Indeed, more than 90% of patients with SM are found to have a somatic gain-of-function mutation in the KIT receptor tyrosine kinase, primarily an aspartic acid to valine substitution (D816V) in the second catalytic domain. This point mutation at molecular level results in enhanced survival and cell autonomous growth of neoplastic MCs [44]. Zappulla et al. generated transgenic mice (by pronuclear injection of the linear Bchm/Asp816Val Kit transgene into fertilized (C57BL/6 DBA2) F2 zygotes) expressing the human D816VKit transgene in MCs to provide a line of evidence for a contribution of D816V mutation in development of mastocytosis. Transgenic mice were harboring a fusion transgene consisting of the 571 bp primate chymase gene (571-bchm) promoter fragment (required for specific expression of the transgene to MCs) in addition to human Kit protooncogene cDNA with the codon 816 Asp → Val substitution. Transgenic and non-transgenic mice were killed at different ages to evaluate the histopathological changes associated with Asp816Val Kit expression. An abnormal accumulation of MCs was accompanied by organ involvement observed in four lines of 12- to 18-month transgenic mice. The spleen as a primary site of MC disease in SM was found to be frequently populated by large MC aggregates in particular within the subcapsular area. Moreover, lymph node and heart involvement were also reported in some transgenic mice. Consistent with the lack of transgene expression in bone marrow, there were no signs of bone marrow involvement. Apparently, this observation was due to use of the chymase promoter, which specifically targets differentiated MCs with expression of mouse MC protease 5. This group of researchers sought to analyze the impact of the Asp816Val Kit mutation on MC proliferation and prepared bone marrow-derived cultured mast cells (BMMCs) from both transgenic and non-transgenic mice. Not surprisingly, only BMMCs from transgenic mice could express the transgene. Transgenic BMMCs could successfully be maintained through continuous cultures for over 24 months. After several months, they became independent of growth factors such as SCF or IL-3. However, exogenous IL-3 was necessary in the initial phases suggesting that, while Asp816Val Kit is necessary for growth factor independent proliferation, other factors are required to confer such ability [45] (Fig. 4). There are other non-specific oncogenic mutations identified recently in patients with mastocytosis including TET2 (TET oncogene family member 2), a putative tumor suppressor gene and N-RAS [5]. Tefferi et al. reported that loss-of-function mutations in TET2 occur at a high frequency in systemic mastocytosis (SM) associated with KITD816V mutations [46]. Although KIT mutations play a key role in the pathogenesis of mastocytosis, they are also the most common additional genetic abnormalities in *t*(8;21) AML with a reported incidence ranging from 26 to 47% [47]. It must be kept in mind that dysregulation of MC apoptosis plays a key role in the pathogenesis of mastocytosis. Interestingly, upregulation of the antiapoptotic protein Bcl-2 in aggressive mastocytosis and also upregulation of another antiapoptotic protein Bcl-X have been reported in bone marrow of patients with ISM [7]. Most recently, the role of programmed cell death protein-1 (PD-1) has been investigated in mastocytosis. The PD-1 receptor is expressed on T and B lymphocytes, while its ligand (PD-L1) is expressed on tumor cells. In addition to various other tumor cells, PD-L1 has been found to be expressed physiologically on MCs and dendritic cells. It had been shown, previously, that tumor cells evade the immune response by abrogating this signaling [48, 49]. Kuklinski et al. investigated skin biopsies from patients with mastocytosis by immunohistochemistry and reported increased expression of PDL1 in MC proliferations [49]. Interestingly, Kataoka et al. reported the expression of PD-1 receptor in clinical samples of human cutaneous mastocytosis and also a human mastocytosis cell line LAD2. PD-1 is categorized as an inhibitory receptor that contains immunoreceptor tyrosine-based inhibitory (ITIM) motifs within its cytosolic domains. It is thought that activation of such inhibitory receptors recruits the non-receptor protein phosphatases, such as SHP-1 or SHP-2, to the ITIMs. The activation of ITIMs followed by reversing the action of tyrosine kinase cascades results in downregulation of AKT [48]. Recently, signaling molecules involved in KIT signaling have been gaining attention as possible therapeutic targets for mastocytosis. For instance, due to its involvement in KIT signaling, AKT has been described as phosphorylated in patients with KIT D816V^+^ SM. Similarly, phosphorylated AKT has been reported in the HMC-1 cell line (human KIT D816V^+^ leukemia MC line) which suggests the involvement of AKT activation in the pathogenesis of mastocytosis [50]. Alterations in KIT mRNA processing have been shown to play a role in SM, as novel KIT transcripts have been detected in aggressive mast cell malignancies [51].

## Symptoms of Mediator Release

**Fig. 5:**
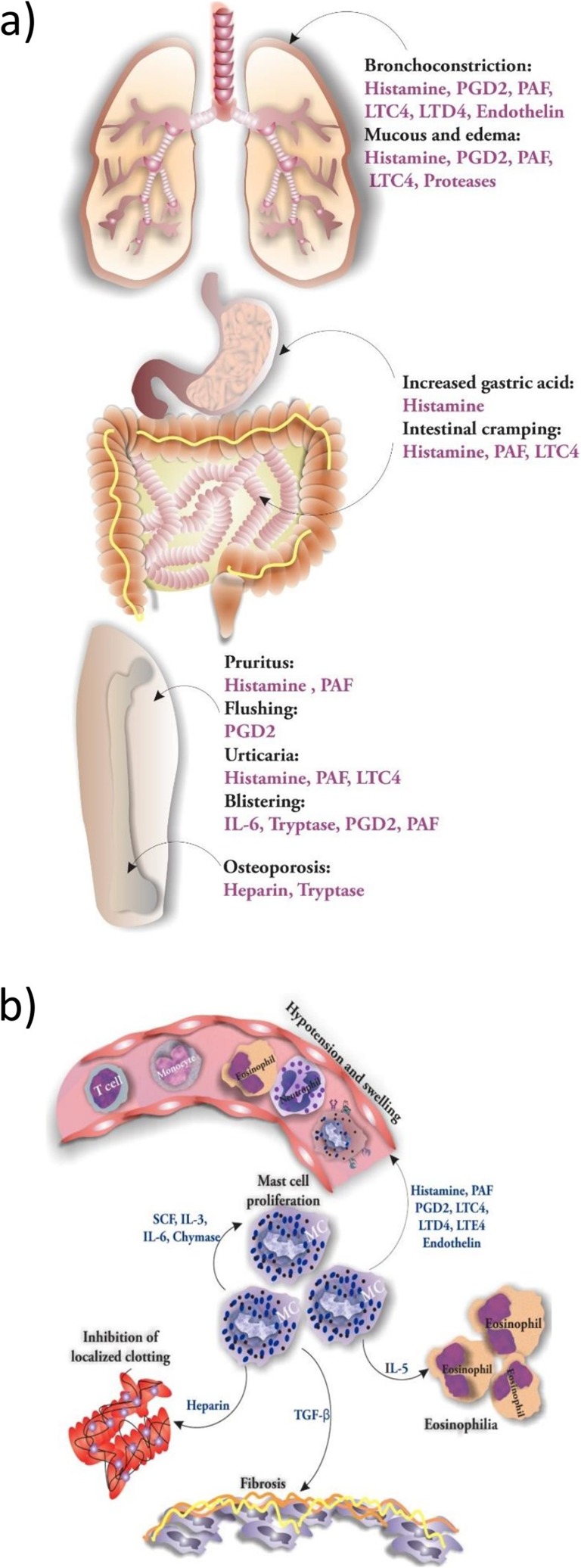
Features associated with release of MC mediators during mastocytosis at two levels of organ involvement (**a**) and cell-cytokine interactions of MCs and tissue cells (**b**)

The symptoms exhibited in mastocytosis are the consequences of increased MC numbers in tissues, and the results of increased mediator release which cause both local and distant effects, when distributed via the circulation. Histamine is the most important mediator acting through four receptors, H1–H4, that mediate vasopermeability, vasodilation, and constriction of bronchial and gastrointestinal smooth muscles, enhancing gastric acid production by parietal cells (via H2 receptors), and pruritus [4]. Generally, H1 receptors control the tone and permeability of the vascular bed, the tone of the intestinal and bronchial smooth muscle, mucus production, heart rate, and flushing responses [52]. Histamine-induced itch is triggered by the excitation of a subset of unmyelinated C-fibers. Neuronal H1 receptors participate in the sensation of itch through activation of phospholipase C. Thus, not surprisingly, H1R blockers (antihistamines) are widely used to manage and alleviate itch symptoms [53]. MC-derived histamine by acting through H1 receptors stimulates fibroblast proliferation and collagen synthesis [54]. Elevated serum tryptase and histamine are general findings in patients with mastocytosis. Upon spontaneous activation of tissue infiltrated MCs, released mediators induce different effects on both tissue residing cells and immune cells [52]. Clinically, these pathophysiologic effects can lead to anaphylaxis. H2 receptors control the vascular permeability, gastric acid secretion, and airway mucus production [55]. H4 receptor is involved in mediating pruritus in mice. The intradermal injection of H4 receptor agonist 4-methylhistamine could induce itch in mice. H4 receptors are expressed in the dorsal root ganglion (DRG) neurons of humans and rats, and their mRNAs have been found in the sensory neurons [56]. Patients with mastocytosis show higher incidence of severe anaphylaxis following hymenoptera stings than in the normal population and baseline serum tryptase should be determined in these patients. A value above 11.4 mg/l is often a clinical clue unmasking an underlying mastocytosis and indicates a high risk of very severe anaphylaxis following re-sting [57]. Not only histamine but also other vasoactive substances such as serotonin (5-HT) and substance P provoke flushing [58]. Chymase is potentiating fibrogenesis by activating TGF-β-induced Smad-dependent pathways. Fibrotic changes in the BM, liver, spleen, and lymph nodes in patients with SM could result from MC released IL-13 and TGF-β [52, 59]. A small proportion of patients with ISM suffer from increased IL-6 levels leading to dysgammaglobulinemia with elevated IgG and IgM levels and monoclonal IgG κ in serum electrophoresis. Tissue eosinophilia may be the result of IL-5 release from MCs. Osteoporosis, osteosclerosis, or osteolysis can be observed in patients with mastocytosis and may be mediated by MC-mass itself as well as IL-1β and IL-6 secreted by MCs [52]. Moreover, heparin and MC-proteases play a role in osteoporosis in patients with mastocytosis [52]. MC cytokines, mainly, TGF-β, FGF, and VEGF, may be associated with tissue remodeling by interacting with endothelium, epithelium, fibroblasts, and macrophages. MCs are a source of IL-31 which is known as a potent mediator of itch. IL-31 levels have been reported to be correlated with disease severity, tryptase levels, and percentages of BM infiltration [60]. Histamine, leukotrienes, endothelin, and PAF cause hypotension and swelling through affecting the endothelial walls of vessels. TGF-β, another MC mediator, induces fibrosis in surrounding tissues [4] (Fig. 5).

## MCAS

The umbrella term mast cell activation disease (MCAD) comprises the full spectrum of primary systemic MC disease, i.e., systemic mastocytosis (SM) which is further divided into the subtypes primary MC activation syndrome (MCAS) and MC leukemia (MCL) [61]. Aberrant release of mast cell mediators is responsible for related symptoms in mast cell activation disorders. Histamine is responsible for the immediate symptoms including headache, hypotension, pruritus, urticaria, angioedema, diarrhea, and anaphylaxis. Symptoms such as cardiac arrhythmias, myocardial infarction, and hypotension may be attributed to aberrant chymase release. Abdominal cramping, pulmonary edema, urticaria, bronchoconstriction, hypotension, arrhythmia, and anaphylaxis are associated with the release of platelet activating factor (PAF). Prostaglandin D2 is responsible for symptoms such as flushing, mucus secretion, bronchoconstriction, vascular instability, headache, nausea, and abdominal pain [62]. Mast cell activation syndromes (MCAS) are a group of disorders that typically present with symptoms of MC mediator release including itching, flushing, whealing, flaring, angioedema, tachycardia, headache, and gastrointestinal manifestations such as abdominal pain and diarrhea [63]. The proposed diagnostic criteria for MCAS include episodic recurrent symptoms consistent with MC activation in more than one organ; decrease in frequency/severity of symptoms in response to MC mediator therapy such as H1 and H2 antihistamines, leukotrienes, cromolyn, and glucocorticoids; and increased MC activation products mainly tryptase above baseline in at least two symptomatic episodes [64]. Other MC products that can be found above normal during MCAS include heparin and chromogranin A in the blood or histamine and its metabolites (e.g., *N*-methylhistamine) in the urine [61]. Lack of distinguishing cardinal signs and symptoms makes it hard to clearly distinguish mastocytosis and MCAS. Widespread distribution of MCs and various pattern of aberrant mediator expression result in a great diversity in the clinical presentation of MCAS. Generally, MCAS does not include the entire body, but may involve a specific organ, such as the bladder or GI tract [65]. Unlike SM, urticaria and angioedema are often present in MCAS. MC shape in bone marrow differs in both diseases in which spindled MCs could be seen in mastocytosis while MCs are round and fully granulated in bone marrow specimens obtained from patients with MCAS [64]. In patients with MCAS, hymenoptera stings, alcohol, and heat are the most common triggers of symptoms [62].

## Clinical Classification

**Fig. 6:**
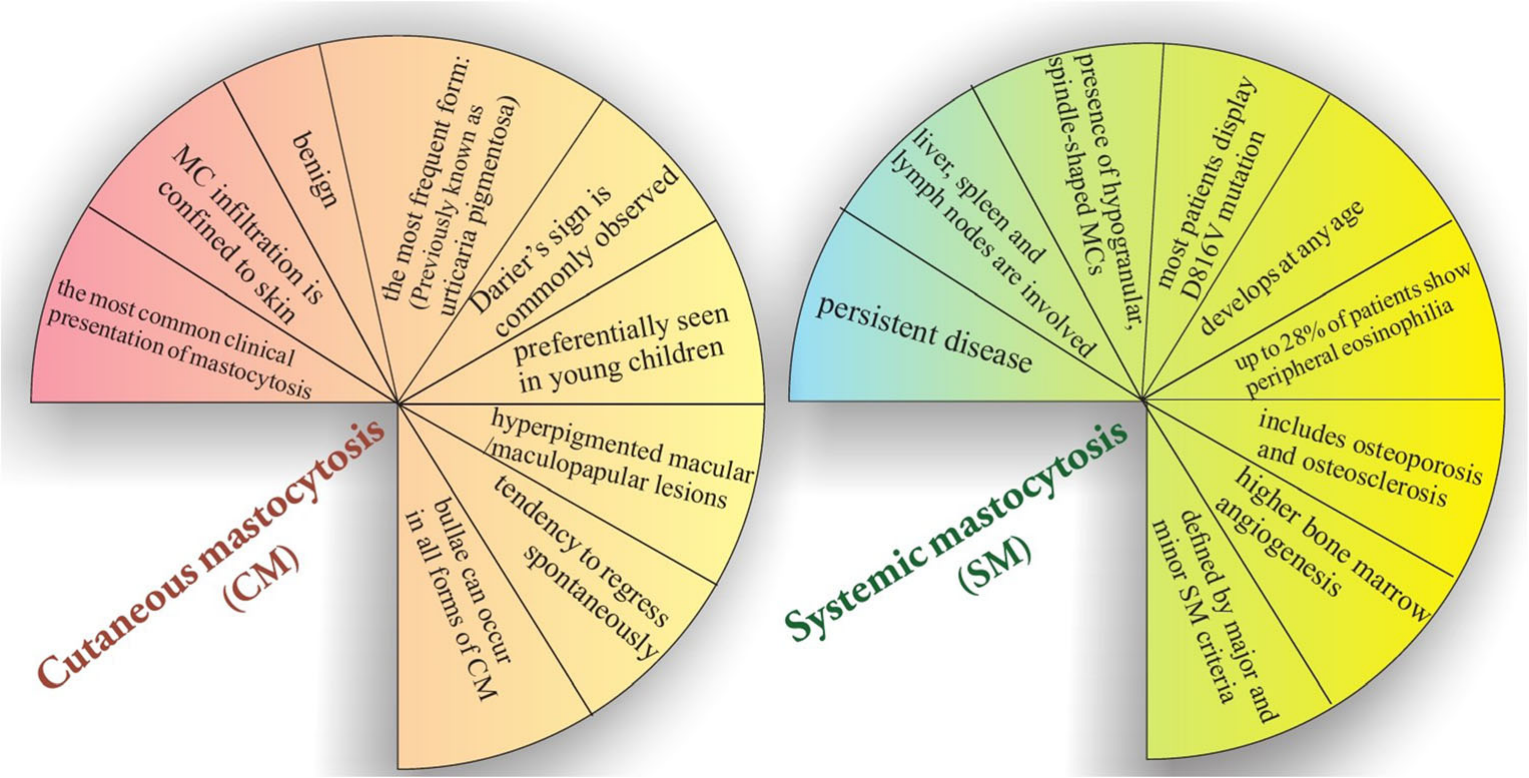
Comparison between CM and SM due to clinical manifestations

Categories of mastocytosis include the following:

1. Cutaneous mastocytosis (CM) which is the most frequent form with favorable prognosis and no organ involvement besides the skin.
2. Systemic mastocytosis (SM) characterized by infiltration of MCs in extracutaneous organs such as the spleen, liver [66], and bone marrow [67]. (Fig. 6)

According to the 2008 World Health Organization (WHO) classification system, mastocytosis can be classified into several subtypes: (1) cutaneous mastocytosis, (2) extracutaneous mastocytosis, (3) mast cell sarcoma, and (4) systemic mastocytosis (SM). SM can be further subdivided into the following subcategories: (1) indolent systemic mastocytosis (ISM); (2) SM associated with another clonal hematological non-mast cell lineage disease (SM-AHNMD), most commonly chronic myelomonocytic leukemia (CMML); (3) aggressive SM (ASM); and (4) mast cell leukemia [68]. (Table 1) The clinical course of patients with SM is highly variable ranging from frequent indolent to rare highly aggressive variants, affecting multiorgan involvement and overall survival. According to WHO, SM is diagnosed when at least one major and one minor or at least three minor SM criteria are fulfilled [7, 69]. (Table 2) Childhood-onset mastocytosis is usually accompanied by a self-limited course, anaphylaxis frequency rates below 10% and a basal serum tryptase level (BST) of <20 μg/l [70]. Children with typical cutaneous lesions usually do not require bone marrow biopsy. However, this procedure may be considered if hepatosplenomegaly, lymphadenopathy, or peripheral-blood abnormalities are observed [71]. BST levels >20 μg/l in pediatric CM have been shown to reflect extensive skin involvement and the possibility of systemic disease [72]. Increased total BST in the absence of acute MC mediator release has long been documented in ISM [73]. Only the small proportion of children who do not remit spontaneously before puberty will experience transformation into SM. According to the most recent classification of CM, proposed by an international task force involving experts from different organizations, adulthood-onset mastocytosis is characterized by a chronic course with 50% prevalence of anaphylaxis [70]. The presence of small monomorphic maculopapular lesions distributed on thigh and trunk is a key feature of this type of CM. In this classification, the typical tryptase level reported is over 20 μg/l and the location of KIT mutation is within exon 17 (most frequently KIT D816V). In case of the presence of a KIT mutation, it is most frequently localized in exon 8, 9, 11, or 17. Large polymorphic maculopapular cutaneous lesions distributed on trunk, head, and extremities are the dominant clinical feature [70]. One of the most important results of this classification is bridging the size of lesions and the age of development with the persistence as a prognostic marker. In this regard, it is suggested that if a monomorphic variant develops in children, it often persists into adulthood, while the polymorphic variant may resolve around puberty [70]. Familial transmission of mastocytosis has been rarely reported since it usually occurs as a result of spontaneous mutation in the C-kit gene [74]. As one of the exceptions to this rule, Wöhrl et al. demonstrated a familial transmission of a mutation in exon 18 at position 849 (S849I) [75].

**Table 1:** WHO systemic mastocytosis variants

Variant term	Subvariants	Features
Cutaneous mastocytosis (CM)		Prevalence ~85% Organ involved: skin [66] Molecular abnormality: KIT D816F, KIT E839K, KIT D816Y [3]
	Urticaria pigmentosa (UP)	Characterized by fixed, reddish brown lesions which occur as maculo-papules, plaques, nodules or blisters [99].
	Maculopapular CM (MPCM) Adulthood-onset mastocytosis Childhood-onset mastocytosis	Small monomorphic lesions Large polymorphic lesions [70, 76]
	Diffuse CM (DCM)	Rare, severe, variant which occurs mainly in infants. Blistering generalized erythroderma, nodules, and plaques are observed [100].
	Skin mastocytoma	
Indolent systemic mastocytosis (ISM)	Smoldering SM	No evidence of organ dysfunction [68]. Therapy: antimediator therapy [3] Prevalence ~10% [5]
	Isolated bone marrow mastocytosis	
Systemic mastocytosis with an associated clonal hematologic non-mast cell Lineage disease (SM-AHNMD)	SM-AML	Prevalence ~1% [5]
	SM-MDS	
	SM-MPD	
	SM-CMML	
	SM-NHL	
Aggressive systemic mastocytosis (ASM)		Impairment of organ function, organomegaly (particularly splenomegaly), cachexia or osteolyses [76]. Therapy: chemotherapy and stem cell transplantation [3] Prevalence ~5% [5]
Mast cell leukemia (MCL)	Aleukemic MCL	Characterized by more than 20% atypical MCs in a bone marrow smear [4, 76]. Very aggressive [68]. Prevalence <1% [5] Therapy: chemotherapy and stem cell transplantation [3]
Mast cell sarcoma		Aggressive neoplasm composed of cytologically malignant MCs presenting as a solitary mass [101]. Prevalence <1% [5]
Extracutaneous mastocytoma		Unifocal MC tumor with low-grade cellular atypia and non-destructive features [68] Prevalence <1% [5]

**Table 2:** Diagnostic criteria for systemic mastocytosis

Major	Multifocal infiltrates of MC in bone marrow sections or other extracutaneous organ(s) (>15 MCs in aggregate)
Minor	• More than 25% of BMMC or MCs of extracutaneous organ(s) are spindle-shaped. • KIT mutation at codon 816 in extracutaneous organ(s) • Presence of CD2+ and/or CD25+ BMMCs • Basal serum tryptase >20 ng/ml (not for patients with AHNMD-type disease)

## Diagnosis

The diagnosis of mastocytosis can be based on the histological examination of a skin biopsy for CM (if necessary and in patients who are suspected clinically in accordance with the recommendation of WHO), and the BM biopsy for the systemic forms according to the recommendation of WHO [76]. The co-expression of KIT and tryptase in MCs in BM makes them easy to detect in histological sections by immunostaining [77]. MCs in SM are characterized by the expression of CD25 and CD2 and an abnormal spindle-shaped hypogranular morphology tending to form clusters around blood vessels and paratrabecular and interstitial areas in the BM [9]. Indolent systemic mastocytosis (ISM) is the least severe systemic variant which is not a life threatening disease [78]. ISM without skin lesions has been frequently reported in those with systemic allergic reactions to hymenoptera venom and raised basal serum tryptase [79]. Gastrointestinal symptoms including abdominal pain, diarrhea, nausea, vomiting, and bloating are found in SM [80]. Up to 28% of patients with SM have peripheral eosinophilia (>650 cells/mm^3^), and this frequency increases in advanced forms [81]. Neoplastic MCs in ASM and MCL, but not ISM, preferentially express CD30 which correlates with a poor overall prognosis [5]. Additionally, myeloproliferative or myelodysplastic syndromes can be observed in patients with SM [71]. Several different staging investigations need to be performed from the BM in patients with SM including microscopic investigations on smears stained with Wright-Giemsa, histology and immunohistochemistry (IHC), cytogenetics, flow cytometry for documenting the expression of CD2 and/or CD25 on neoplastic MCs, and PCR to detect KIT D816V [3, 82]. Generally, the life expectancy in patients with SM depends on the diagnosed variant in which indolent forms do not shorten life expectancy whereas advanced SM variants, including MCL, SM-AHNMD, and ASM have survival rates ranging from months to a few years despite cytoreductive therapy [83].

Approximately 40% suffer from SM. SM may be associated with other hematologic neoplasms not confined to the MC lineage [84]. For example, Tschandl et al. described in a case report a 61-year-old patient with three diseases occurring synchronously: CMML, xanthogranulomas, and systemic mastocytosis [85]. Coincidence of SM with other diseases such as pulmonary interstitial disease [86], refractory pruritus and cirrhosis [87], and Kounis syndrome [88] has been documented in case reports. New potential biomarkers for predicting episodes of mediator release and monitoring the treatment were described recently. For example, Ehara et al. found the Melanoma inhibitory activity (MIA), an 11-kD protein used as a serum marker for malignant melanoma, to be elevated in children with CM [89]. Not only serum biomarkers but also MC surface markers may be of importance in the diagnosis and classification of the disease. Although co-expression of CD2 and CD25 has been known as a typical feature of neoplastic MCs for several decades, recently also other markers such as CD63, CD69, CD58, CD33, and several complement-associated molecules such as CD11c and CD35 have been found to be overexpressed in these cells. Immunophenotyping of MCs using these markers through flow cytometry revealed that MCs in ASM are of the CD25^+^CD2^−^CD63^+^CD69^+^ whereas MCs in MCL of the CD25^+^CD2^−^CD63^−^CD69^−^ phenotype [68].

## Treatment

Control of the immediate, possibly severe symptoms is a common component in disease management of mastocytosis regardless of the subtype. For instance, H1-antihistamines are commonly used for the reduction of pruritus and flushing, H2-antihistamines to treat gastrointestinal (GI) symptoms, and corticosteroids and/or analgesics for mitigating bone pain and other symptoms [90]. Cromoglicinic acid is only weakly effective but may act as adjuvant MC stabilizer through reducing the calcium influx for MC degranulation following FcεR1 crosslinking especially for GI symptoms. Furthermore, ketotifen acts in the same way as other H1-antihistamines because of its stabilizing effects. Omalizumab (Xolair®, Novartis), a humanized murine monoclonal antibody with ability to conjugate in vivo with free serum IgE, is able to reduce binding to FCεRI on MCs and basophils and can be used as an additional measure for controlling the immediate symptoms of MC activation [91]. Oral glucocorticoids, through decreasing the number of connective tissue MCs in a dose-dependent fashion, inhibit SCF production and decrease FcεR1 expression and chemokine receptors including CCR3. Acetylic salicylic acid (Aspirin®, Bayer), a non-steroidal antiinflammatory drug (NSAID), has positive effects in patients with mastocytosis by normalizing the levels of PGD2 metabolites through irreversible inhibition of the cyclooxygenase (COX) isoenzymes. Interferon-α (IFN-α) and cladribine (2-CdA) are used in patients with aggressive SM. IFN-α acts through several mechanisms including decreasing MC mediator release, organ infiltration and normalization of serum tryptase level [9]. Imatinib mesylate (Gleevec®, Novartis) is an ATP-competitive, orally bioavailable agent and the only FDA-approved inhibitor of various tyrosine kinases including ABL1, platelet-derived growth factor receptor (PDGFR), ARG, and KIT for use in patients with ASM without D816V KIT or wild type KIT or sporadic KIT mutant isoforms in SM, such as KIT F522C [66, 92]. Moreover, it has therapeutic applications in chronic myelogenous leukemia and gastrointestinal stromal tumors [93, 94]. Other tyrosine kinase inhibitors include Nilotinib (targets Breakpoint Cluster Region-Abelson (BCR-ABL), KIT, and PDGFR), Dasatinib (targets BCR-ABL, SRC, and KIT), Masitinib (multitargeted inhibitor of the KIT, PDGFR, fibroblast growth factor receptor 3, and Lyn tyrosine kinases), and Midostaurin also known as PKC412 (inhibitor of KIT, fms-related tyrosine kinase 3, vascular endothelial growth factor receptor 2, and PDGFR) [66]. Clinical trials are investigating treatment particularly with KIT D816V inhibitors to be approved officially by FDA [95]. Furthermore, narrowband ultraviolet (UVB) phototherapy is an alternative treatment option in patients with CM [96]. Ustun et al. investigated the effectiveness of allogeneic hematopoietic stem-cell transplantation (alloHCT) in patients with advanced SM, who had undergone either sibling or unrelated alloHCT. Three parameters were used to assess response before and after transplantation including the percentage of bone marrow MCs, serum tryptase levels, and organ involvement. The median bone marrow MC percentage in biopsies and the levels of serum tryptase showed a significant decrease after transplantation. They reported that alloHCT can confer long-term overall survival [83]. Baumgartner et al. showed that neoplastic MCs in patients with SM exhibit phosphorylated STAT5 (pSTAT5) in cytoplasm as an essential growth/survival factor, so targeting of pSTAT5 apparently could be an approach in treatment of SM [97]. Moreover, Sharma et al. investigated the role of SHP2/PTPN11 phosphatase in oncogenic KIT signaling using an aggressive SM mouse model. They reported that stable knockdown of SHP2 results in impaired growth, colony formation, and increased rates of apoptosis in mouse mastocytoma cell line P815 harboring KITD^814Y^ mutation [98].

## Conclusion

Mastocytosis is a group of rare clonal disorders characterized by abnormal expansion and accumulation of tissue MCs in one or multiple organs. Trafficking patterns of neoplastic MCs within the BM and also augmented angiogenesis in the BM during SM in particular are interesting aspects of this disease and should be addressed in further investigations. The clonal nature of the disease can be established through the demonstration of gain-of-function mutations involving the tyrosine kinase domain of KIT receptor in skin and/or BM cells. The heterogeneity of clinical presentations of mastocytosis relates to the tissue MC burden. There is much variation in the type of skin lesions, the patient’s age at the onset, and associated hematological disorders that—taken together—make the treatment of the disease challenging. The clinical symptoms are mediated by the release of MC mediators. Management of patients within all categories of mastocytosis includes avoidance of triggering factors such as allergen. Additionally, continuous training for the correct application of the rescue self-medication (including self-injectable intramuscular epinephrine and, as warranted, antihistamine and corticosteroids) for patients and children that are at increased risk of anaphylaxis is constantly required. In recent years, various tyrosine kinase inhibitors have also been employed in order to reduce the MC-load for SM. However, currently, there is no approved tyrosine kinase inhibitor to inhibit the D816V c-kit mutation for routine settings. Moreover, most drugs including KIT D816V blocking agents have not demonstrated a promising efficacy in achieving a long-lasting remission in patients with advanced SM.

## Abbreviations

MAdCAM-1: Mucosal addressin cell adhesion molecule-1
VCAM-1: Vascular cell adhesion molecule-1
NGF: Nerve growth factor
SM: Systemic mastocytosis
CM: Cutaneous mastocytosis
MC: Mast cell
SCF: Stem cell factor
ISM: Indolent systemic mastocytosis
BST: Baseline serum tryptase
alloHCT: Allogeneic hematopoietic stem cell transplantation
TK: Tyrosine kinase

## References

[1] Reber LL, Sibilano R, Mukai K, Galli SJ (2015). Potential effector and immunoregulatory functions of mast cells in mucosal immunity. Mucosal Immunol.

[2] Ronnberg E, Melo FR, Pejler G (2012). Mast cell proteoglycans. J Histochem Cytochem Off J Histochem Soc.

[3] Valent P (2013). Mastocytosis: a paradigmatic example of a rare disease with complex biology and pathology. Am J Cancer Res.

[4] Carter MC, Metcalfe DD, Komarow HD (2014). Mastocytosis. Immunol Allergy Clin N Am.

[5] Magliacane D, Parente R, Triggiani M (2014). Current concepts on diagnosis and treatment of mastocytosis. Translat Med @ UniSa.

[6] Kushnir-Sukhov NM, Brittain E, Scott L, Metcalfe DD (2008). Clinical correlates of blood serotonin levels in patients with mastocytosis. Eur J Clin Investig.

[7] Lange M, Nedoszytko B, Gorska A, Zawrocki A, Sobjanek M, Kozlowski D (2012). Mastocytosis in children and adults: clinical disease heterogeneity. Arch Med Sci.

[8] Gilfillan AM, Austin SJ, Metcalfe DD (2011). Mast cell biology: introduction and overview. Adv Exp Med Biol.

[9] Cardet JC, Akin C, Lee MJ (2013). Mastocytosis: update on pharmacotherapy and future directions. Expert Opin Pharmacother.

[10] Galli SJ, Starkl P, Marichal T, Tsai M (2016). Mast cells and IgE in defense against venoms: possible “good side” of allergy?. Allergology Int Off J Jpn Soc Allergol.

[11] Elieh Ali Komi D, Shafaghat F, Zwiener RD (2017) Immunology of bee venom. Clin Rev Allergy Immunol. doi:10.1007/s12016-017-8597-4 10.1007/s12016-017-8597-4 28105558

[12] Overed-Sayer C, Rapley L, Mustelin T, Clarke DL (2013). Are mast cells instrumental for fibrotic diseases?. Front Pharmacol.

[13] Theoharides TC, Alysandratos KD, Angelidou A, Delivanis DA, Sismanopoulos N, Zhang B, Asadi S, Vasiadi M, Weng Z, Miniati A, Kalogeromitros D (2012). Mast cells and inflammation. Biochim Biophys Acta.

[14] Galli SJ, Tsai M (2008). Mast cells: versatile regulators of inflammation, tissue remodeling, host defense and homeostasis. J Dermatol Sci.

[15] Okayama Y, Kawakami T (2006). Development, migration, and survival of mast cells. Immunol Res.

[16] Dahlin JS, Ding Z, Hallgren J (2015). Distinguishing mast cell progenitors from mature mast cells in mice. Stem Cells Dev.

[17] Elieh-Ali-Komi D, Cao Y (2016) Role of mast cells in the pathogenesis of multiple sclerosis and experimental autoimmune encephalomyelitis. Clin Rev Allergy Immunol. doi:10.1007/s12016-016-8595-y 10.1007/s12016-016-8595-y 28025778

[18] Halova I, Draberova L, Draber P (2012). Mast cell chemotaxis—chemoattractants and signaling pathways. Front Immunol.

[19] Merz H, Kaehler C, Hoefig KP, Branke B, Uckert W, Nadrowitz R, Cerny-Reiterer S, Herrmann H, Feller AC, Valent P (2010). Interleukin-9 (IL-9) and NPM-ALK each generate mast cell hyperplasia as single ‘hit’ and cooperate in producing a mastocytosis-like disease in mice. Oncotarget.

[20] Migalovich-Sheikhet H, Friedman S, Mankuta D, Levi-Schaffer F (2012). Novel identified receptors on mast cells. Front Immunol.

[21] da Silva EZ, Jamur MC, Oliver C (2014). Mast cell function: a new vision of an old cell. J Histochem Cytochem Off J Histochem Soc.

[22] Puri N, Roche PA (2008). Mast cells possess distinct secretory granule subsets whose exocytosis is regulated by different SNARE isoforms. Proc Natl Acad Sci U S A.

[23] Wasiuk A, de Vries VC, Hartmann K, Roers A, Noelle RJ (2009). Mast cells as regulators of adaptive immunity to tumours. Clin Exp Immunol.

[24] Voehringer D (2013). Protective and pathological roles of mast cells and basophils. Nat Rev Immunol.

[25] Lundequist A, Pejler G (2011). Biological implications of preformed mast cell mediators. Cell Mol Life Sci.

[26] Moon TC, Befus AD, Kulka M (2014). Mast cell mediators: their differential release and the secretory pathways involved. Front Immunol.

[27] Gri G, Frossi B, D'Inca F, Danelli L, Betto E, Mion F, Sibilano R, Pucillo C (2012). Mast cell: an emerging partner in immune interaction. Front Immunol.

[28] Rigoni A, Colombo MP, Pucillo C (2015). The role of mast cells in molding the tumor microenvironment. Cancer Microenviron Off J Int Cancer Microenviron Soc.

[29] Schafer B, Piliponsky AM, Oka T, Song CH, Gerard NP, Gerard C, Tsai M, Kalesnikoff J, Galli SJ (2013). Mast cell anaphylatoxin receptor expression can enhance IgE-dependent skin inflammation in mice. The Journal of allergy and clinical immunology.

[30] Balseiro-Gomez S, Flores JA, Acosta J, Ramirez-Ponce MP, Ales E (2015). Identification of a new exo-endocytic mechanism triggered by corticotropin-releasing hormone in mast cells. J Immunol (Baltimore, Md: 1950).

[31] Ciprandi G, Marseglia GL, Castagnoli R, Valsecchi C, Tagliacarne C, Caimmi S, Licari A (2015). From IgE to clinical trials of allergic rhinitis. Expert Rev Clin Immunol.

[32] Rivera J, Fierro NA, Olivera A, Suzuki R (2008). New insights on mast cell activation via the high affinity receptor for IgE. Adv Immunol.

[33] Shin JS, Greer AM (2015). The role of FcepsilonRI expressed in dendritic cells and monocytes. Cell Mol Life Sci.

[34] Ashman LK (1999). The biology of stem cell factor and its receptor C-kit. Int J Biochem Cell Biol.

[35] Geissler EN, Liao M, Brook JD, Martin FH, Zsebo KM, Housman DE, Galli SJ (1991). Stem cell factor (SCF), a novel hematopoietic growth factor and ligand for c-kit tyrosine kinase receptor, maps on human chromosome 12 between 12q14.3 and 12qter. Somat Cell Mol Genet.

[36] Bai CG, Hou XW, Wang F, Qiu C, Zhu Y, Huang L, Zhao J, Xu JJ, Ma DL (2012). Stem cell factor-mediated wild-type KIT receptor activation is critical for gastrointestinal stromal tumor cell growth. World J Gastroenterol.

[37] Cruse G, Metcalfe DD, Olivera A (2014). Functional deregulation of KIT: link to mast cell proliferative diseases and other neoplasms. Immunol Allergy Clin N Am.

[38] Besmer P, Manova K, Duttlinger R, Huang EJ, Packer A, Gyssler C, Bachvarova RF (1993) The kit-ligand (steel factor) and its receptor c-kit/W: pleiotropic roles in gametogenesis and melanogenesis. Development (Cambridge, England) Supplement:125–137 7519481

[39] Moore S, McDiarmid LA, Hughes TP (2000). Stem cell factor and chronic myeloid leukemia CD34+ cells. Leuk Lymphoma.

[40] Lennartsson J, Ronnstrand L (2012). Stem cell factor receptor/c-Kit: from basic science to clinical implications. Physiol Rev.

[41] Wang X, Ren H, Zhao T, Chen J, Sun W, Sun Y, Ma W, Wang J, Gao C, Gao S, Lang M, Jia L, Hao J (2014). Stem cell factor is a novel independent prognostic biomarker for hepatocellular carcinoma after curative resection. Carcinogenesis.

[42] Metcalfe DD (2008). Mast cells and mastocytosis. Blood.

[43] Morales JK, Falanga YT, Depcrynski A, Fernando J, Ryan JJ (2010). Mast cell homeostasis and the JAK-STAT pathway. Genes Immun.

[44] Chatterjee A, Ghosh J, Kapur R (2015). Mastocytosis: a mutated KIT receptor induced myeloproliferative disorder. Oncotarget.

[45] Zappulla JP, Dubreuil P, Desbois S, Letard S, Hamouda NB, Daeron M, Delsol G, Arock M, Liblau RS (2005). Mastocytosis in mice expressing human Kit receptor with the activating Asp816Val mutation. J Exp Med.

[46] Tefferi A, Levine RL, Lim KH, Abdel-Wahab O, Lasho TL, Patel J, Finke CM, Mullally A, Li CY, Pardanani A, Gilliland DG (2009). Frequent TET2 mutations in systemic mastocytosis: clinical, KITD816V and FIP1L1-PDGFRA correlates. Leukemia.

[47] Pullarkat ST, Pullarkat V, Kroft SH, Wilson CS, Ahsanuddin AN, Mann KP, Thein M, Grody WW, Brynes RK (2009). Systemic mastocytosis associated with t(8;21)(q22;q22) acute myeloid leukemia. J Hematop.

[48] Kataoka TR, Fujimoto M, Moriyoshi K, Koyanagi I, Ueshima C, Kono F, Tsuruyama T, Okayama Y, Ra C, Haga H (2013). PD-1 regulates the growth of human mastocytosis cells. Allergol Int Off J Jpn Soc Allergol.

[49] Kuklinski LF, Kim J (2016). Expression of PD-L1 in mastocytosis. J Am Acad Dermatol.

[50] Bibi S, Arslanhan MD, Langenfeld F, Jeanningros S, Cerny-Reiterer S, Hadzijusufovic E, Tchertanov L, Moriggl R, Valent P, Arock M (2014). Co-operating STAT5 and AKT signaling pathways in chronic myeloid leukemia and mastocytosis: possible new targets of therapy. Haematologica.

[51] Chan EC, Bai Y, Bandara G, Simakova O, Brittain E, Scott L, Dyer KD, Klion AD, Maric I, Gilfillan AM, Metcalfe DD, Wilson TM (2013). KIT GNNK splice variants: expression in systemic mastocytosis and influence on the activating potential of the D816V mutation in mast cells. Experimental hematology.

[52] Castells M, Austen KF (2002). Mastocytosis: mediator-related signs and symptoms. International archives of allergy and immunology.

[53] Shim WS, Oh U (2008). Histamine-induced itch and its relationship with pain. Mol Pain.

[54] Yang L, Murota H, Serada S, Fujimoto M, Kudo A, Naka T, Katayama I (2014). Histamine contributes to tissue remodeling via periostin expression. J Invest Dermatol.

[55] Castells M (2006). Mast cell mediators in allergic inflammation and mastocytosis. Immunol Allergy Clin N Am.

[56] Jian T, Yang N, Yang Y, Zhu C, Yuan X, Yu G, Wang C, Wang Z, Shi H, Tang M, He Q, Lan L, Wu G, Tang Z (2016). TRPV1 and PLC participate in histamine H4 receptor-induced itch. Neural Plast.

[57] Bonadonna P, Zanotti R, Muller U (2010). Mastocytosis and insect venom allergy. Curr Opin Allergy Clin Immunol.

[58] Hannah-Shmouni F, Stratakis CA, Koch CA (2016). Flushing in (neuro)endocrinology. Rev Endocr Metab Disord.

[59] Sukrithan VK, Salamon JN, Berulava G, Sibinga NE, Verma A (2016). Systemic mastocytosis presenting as cardiac tamponade with CD25(+) pericardial mast cells. Clin Case Rep.

[60] Arock M, Akin C, Hermine O, Valent P (2015). Current treatment options in patients with mastocytosis: status in 2015 and future perspectives. Eur J Haematol.

[61] Molderings GJ, Haenisch B, Brettner S, Homann J, Menzen M, Dumoulin FL, Panse J, Butterfield J, Afrin LB (2016). Pharmacological treatment options for mast cell activation disease. Naunyn Schmiedeberg's Arch Pharmacol.

[62] Picard M, Giavina-Bianchi P, Mezzano V, Castells M (2013). Expanding spectrum of mast cell activation disorders: monoclonal and idiopathic mast cell activation syndromes. Clin Ther.

[63] Nurmatov UB, Rhatigan E, Simons FE, Sheikh A (2015). H1-antihistamines for primary mast cell activation syndromes: a systematic review. Allergy.

[64] Akin C (2015). Mast cell activation syndromes presenting as anaphylaxis. Immunol Allergy Clin N Am.

[65] Ratner V (2015). Mast cell activation syndrome. Transl Androl Urol.

[66] Verstovsek S (2013). Advanced systemic mastocytosis: the impact of KIT mutations in diagnosis, treatment, and progression. Eur J Haematol.

[67] Fernandes IC, Teixeira Mdos A, Freitas I, Selores M, Alves R, Lima M (2014). Adult mastocytosis: a review of the Santo Antonio Hospital’s experience and an evaluation of World Health Organization criteria for the diagnosis of systemic disease. An Bras Dermatol.

[68] Quintas-Cardama A, Jain N, Verstovsek S (2011). Advances and controversies in the diagnosis, pathogenesis, and treatment of systemic mastocytosis. Cancer.

[69] Sanchez-Munoz L, Alvarez-Twose I, Garcia-Montero AC, Teodosio C, Jara-Acevedo M, Pedreira CE, Matito A, Morgado JM, Sanchez ML, Mollejo M, Gonzalez-de-Olano D, Orfao A, Escribano L (2011). Evaluation of the WHO criteria for the classification of patients with mastocytosis. Mod Pathol Off J US Can Acad Pathol Inc.

[70] Hartmann K, Escribano L, Grattan C, Brockow K, Carter MC, Alvarez-Twose I, Matito A, Broesby-Olsen S, Siebenhaar F, Lange M, Niedoszytko M, Castells M, Oude Elberink JN, Bonadonna P, Zanotti R, Hornick JL, Torrelo A, Grabbe J, Rabenhorst A, Nedoszytko B, Butterfield JH, Gotlib J, Reiter A, Radia D, Hermine O, Sotlar K, George TI, Kristensen TK, Kluin-Nelemans HC, Yavuz S, Hagglund H, Sperr WR, Schwartz LB, Triggiani M, Maurer M, Nilsson G, Horny HP, Arock M, Orfao A, Metcalfe DD, Akin C, Valent P (2016). Cutaneous manifestations in patients with mastocytosis: consensus report of the European Competence Network on Mastocytosis; the American Academy of Allergy, Asthma & Immunology; and the European Academy of Allergology and Clinical Immunology. J Allergy Clin Immunol.

[71] Theoharides TC, Valent P, Akin C (2015). Mast cells, mastocytosis, and related disorders. N Engl J Med.

[72] Alvarez-Twose I, Vano-Galvan S, Sanchez-Munoz L, Morgado JM, Matito A, Torrelo A, Jaen P, Schwartz LB, Orfao A, Escribano L (2012). Increased serum baseline tryptase levels and extensive skin involvement are predictors for the severity of mast cell activation episodes in children with mastocytosis. Allergy.

[73] Matito A, Morgado JM, Alvarez-Twose I, Sanchez-Munoz L, Pedreira CE, Jara-Acevedo M, Teodosio C, Sanchez-Lopez P, Fernandez-Nunez E, Moreno-Borque R, Garcia-Montero A, Orfao A, Escribano L (2013). Serum tryptase monitoring in indolent systemic mastocytosis: association with disease features and patient outcome. PLoS One.

[74] Tuysuz G, Ozdemir N, Apak H, Kutlubay Z, Demirkesen C, Celkan T (2015). Childhood mastocytosis: results of a single center. Turk pediatri arsivi.

[75] Wöhrl S, Moritz KB, Bracher A, Fischer G, Stingl G, Loewe R (2013). A c-kit mutation in exon 18 in familial mastocytosis. J Invest Dermatol.

[76] Horny HP, Sotlar K, Valent P, Hartmann K (2008). Mastocytosis: a disease of the hematopoietic stem cell. Dtsch Arztebl Int.

[77] Sotlar K, Cerny-Reiterer S, Petat-Dutter K, Hessel H, Berezowska S, Mullauer L, Valent P, Horny HP (2011). Aberrant expression of CD30 in neoplastic mast cells in high-grade mastocytosis. Mod Pathol Off J US Can Acad Pathol Inc.

[78] Moura DS, Sultan S, Georgin-Lavialle S, Barete S, Lortholary O, Gaillard R, Hermine O (2012). Evidence for cognitive impairment in mastocytosis: prevalence, features and correlations to depression. PLoS One.

[79] Zanotti R, Bonadonna P, Bonifacio M, Artuso A, Schena D, Rossini M, Perbellini O, Colarossi S, Chilosi M, Pizzolo G (2011). Isolated bone marrow mastocytosis: an underestimated subvariant of indolent systemic mastocytosis. Haematologica.

[80] Lee JK, Whittaker SJ, Enns RA, Zetler P (2008). Gastrointestinal manifestations of systemic mastocytosis. World J Gastroenterol.

[81] Kovalszki A, Weller PF (2014). Eosinophilia in mast cell disease. Immunol Allergy Clin N Am.

[82] Morgado JM, Sanchez-Munoz L, Teodosio CG, Jara-Acevedo M, Alvarez-Twose I, Matito A, Fernandez-Nunez E, Garcia-Montero A, Orfao A, Escribano L (2012). Immunophenotyping in systemic mastocytosis diagnosis: ‘CD25 positive’ alone is more informative than the ‘CD25 and/or CD2’ WHO criterion. Mod Pathol Off J US Can Acad Pathol Inc.

[83] Ustun C, Reiter A, Scott BL, Nakamura R, Damaj G, Kreil S, Shanley R, Hogan WJ, Perales MA, Shore T, Baurmann H, Stuart R, Gruhn B, Doubek M, Hsu JW, Tholouli E, Gromke T, Godley LA, Pagano L, Gilman A, Wagner EM, Shwayder T, Bornhauser M, Papadopoulos EB, Bohm A, Vercellotti G, Van Lint MT, Schmid C, Rabitsch W, Pullarkat V, Legrand F, Yakoub-Agha I, Saber W, Barrett J, Hermine O, Hagglund H, Sperr WR, Popat U, Alyea EP, Devine S, Deeg HJ, Weisdorf D, Akin C, Valent P (2014). Hematopoietic stem-cell transplantation for advanced systemic mastocytosis. J Clin Oncol Off J Am Soc Clin Oncol.

[84] Zhrebker L, Cooper B, Krause JR (2014). Systemic mastocytosis with associated acute myelogenous leukemia. Proc (Baylor Univ Med Cent).

[85] Tschandl P, Mullauer L, Kittler H (2012). Systemic mastocytosis associated with chronic myelomonocytic leukemia and xanthogranuloma. Dermatology practical & conceptual.

[86] Hermans MA, Broijl A, van Daele PL (2016). A unique presentation of pulmonary disease in advanced systemic mastocytosis, proven by the presence of mast cells in bronchoalveolar lavage: a case report. J Med Case Rep.

[87] Addepally NS, Klair JS, Girotra M, Jones J, Aduli F (2016). Systemic mastocytosis causing refractory pruritus in a liver disease patient. ACG case reports journal.

[88] Lerner M, Pal RS, Borici-Mazi R (2017). Kounis syndrome and systemic mastocytosis in a 52-year-old man having surgery. CMAJ: Canadian Medical Association journal = journal de l’Association medicale canadienne.

[89] Ehara Y, Yoshida Y, Tahira M, Yamamoto O (2014). The expression of melanoma inhibitory activity on mast cells in child patients with cutaneous mastocytosis. Yonago Acta Med.

[90] Hochhaus A, Baccarani M, Giles FJ, le Coutre PD, Muller MC, Reiter A, Santanastasio H, Leung M, Novick S, Kantarjian HM (2015). Nilotinib in patients with systemic mastocytosis: analysis of the phase 2, open-label, single-arm nilotinib registration study. J Cancer Res Clin Oncol.

[91] Sokol KC, Ghazi A, Kelly BC, Grant JA (2014). Omalizumab as a desensitizing agent and treatment in mastocytosis: a review of the literature and case report. J Allergy Clin Immunol Pract.

[92] Vega-Ruiz A, Cortes JE, Sever M, Manshouri T, Quintas-Cardama A, Luthra R, Kantarjian HM, Verstovsek S (2009). Phase II study of imatinib mesylate as therapy for patients with systemic mastocytosis. Leuk Res.

[93] Napier RJ, Norris BA, Swimm A, Giver CR, Harris WA, Laval J, Napier BA, Patel G, Crump R, Peng Z, Bornmann W, Pulendran B, Buller RM, Weiss DS, Tirouvanziam R, Waller EK, Kalman D (2015). Low doses of imatinib induce myelopoiesis and enhance host anti-microbial immunity. PLoS Pathog.

[94] Pereira PR, Odashiro AN, Marshall JC, Correa ZM, Belfort R, Burnier MN (2005). The role of c-kit and imatinib mesylate in uveal melanoma. J Carcinog.

[95] Yacoub A, Prochaska L (2016). Ruxolitinib improves symptoms and quality of life in a patient with systemic mastocytosis. Biomarker Res.

[96] Prignano F, Troiano M, Lotti T (2010). Cutaneous mastocytosis: successful treatment with narrowband ultraviolet B phototherapy. Clin Exp Dermatol.

[97] Baumgartner C, Cerny-Reiterer S, Sonneck K, Mayerhofer M, Gleixner KV, Fritz R, Kerenyi M, Boudot C, Gouilleux F, Kornfeld JW, Sillaber C, Moriggl R, Valent P (2009). Expression of activated STAT5 in neoplastic mast cells in systemic mastocytosis: subcellular distribution and role of the transforming oncoprotein KIT D816V. Am J Pathol.

[98] Sharma N, Everingham S, Zeng LF, Zhang ZY, Kapur R, Craig AW (2014). Oncogenic KIT-induced aggressive systemic mastocytosis requires SHP2/PTPN11 phosphatase for disease progression in mice. Oncotarget.

[99] Ferrante G, Scavone V, Muscia MC, Adrignola E, Corsello G, Passalacqua G, La Grutta S (2015). The care pathway for children with urticaria, angioedema, mastocytosis. World Allergy Organ J.

[100] Edwards AM, Capkova S (2011) Oral and topical sodium cromoglicate in the treatment of diffuse cutaneous mastocytosis in an infant. BMJ Case Rep. doi:10.1136/bcr.02.2011.3910 10.1136/bcr.02.2011.3910 PMC3128352 22693187

[101] Ryan RJ, Akin C, Castells M, Wills M, Selig MK, Nielsen GP, Ferry JA, Hornick JL (2013). Mast cell sarcoma: a rare and potentially under-recognized diagnostic entity with specific therapeutic implications. Mod Pathol Off J US Can Acad Pathol Inc.

